# A midline retroperitoneal approach for complex abdominal aortic repair: Case description and operative technique

**DOI:** 10.1016/j.jvscit.2022.08.030

**Published:** 2022-09-28

**Authors:** EeeLN Buckarma, Jason Beckermann, Carmelina Gurrieri, Brett Frodl, Nishant Saran, Thomas Carmody, Tiziano Tallarita

**Affiliations:** a Department of Surgery, Mayo Clinic, Rochester, MN; b Department of Cardiovascular Surgery, Mayo Clinic, Eau Claire, WI; c Department of Anesthesiology, Mayo Clinic, Eau Claire, WI

**Keywords:** Abdominal aortic aneurysm, Aortic atherosclerotic disease, Midline retroperitoneal repair, Operative technique

## Abstract

In the current endovascular era, open repair of complex aortic aneurysms is becoming a rare, but indispensable, part of vascular surgeons’ skill set in specific scenarios. For young, low-risk patients and patients with connective tissue disorders, early target vessel bifurcation, a horseshoe kidney, or pedunculated intraluminal aortic thrombus, fenestrated-branched stent graft technology will not be applicable without significant risks. Thus, an open surgical approach has been recommended for these patients. Most vascular surgeons will be familiar with a transperitoneal approach or a retroperitoneal approach with a lateral incision. For patients with a horseshoe kidney, an inflammatory aneurysm, or a history of multiple intraperitoneal procedures, a retroperitoneal approach should be preferred. In the present report, we have described in detail the optimization of a retroperitoneal approach through a midline incision that provides excellent exposure to the paravisceral aorta, improves exposure to the right renal artery and right iliac artery bifurcation (which is limited using the left flank retroperitoneal approach), and avoids division of the lateral abdominal wall muscles, which has often been associated with iatrogenic muscle denervation and postoperative bulging for four patients who had required complex aortic reconstruction.

Endovascular repair of infrarenal aortic aneurysms has become the first-line therapy for selected patients with suitable anatomy owing to the shorter hospital stays, decreased blood transfusion requirements, and reduced early morbidity and mortality compared with open repair.^1^ Complex abdominal aortic aneurysms, defined as those that require, at a minimum, suprarenal clamping, have traditionally been repaired via an open approach. However, in the past decade, endovascular repair with fenestrated branch technology has become an important alternative for patients with suitable anatomy.^1^ Among the open approaches, transperitoneal repair for complex abdominal aortic aneurysms has the advantage of being familiar to most vascular and general surgeons and allows for access to the suprarenal aorta and bilateral iliac bifurcation. Using a left medial visceral rotation, the surgeon can also access the supraceliac aorta, if needed. In specific scenarios, such as a horseshoe kidney, an inflammatory aneurysm, previous intraperitoneal abdominal surgery, retroperitoneal exposure through a flank incision could be preferred.^2^ However, this approach provides limited or no access to the right renal artery and right iliac bifurcation and can be associated with increased postoperative pain and a bulging flank from iatrogenic lateral abdominal wall muscle denervation. The midline retroperitoneal approach (MRA) has been described, although only for infrarenal aortic disease, usually as an alternative option to the retroperitoneal approach (RPA) to avoid division of the abdominal muscles. In the present report, we have described in detail a further development of the RPA through a midline incision to treat complex abdominal aortic disease that improves exposure to the right renal artery and right iliac artery bifurcation and maintains excellent access to the paravisceral aorta.

## Technique description

**Fig 1:**
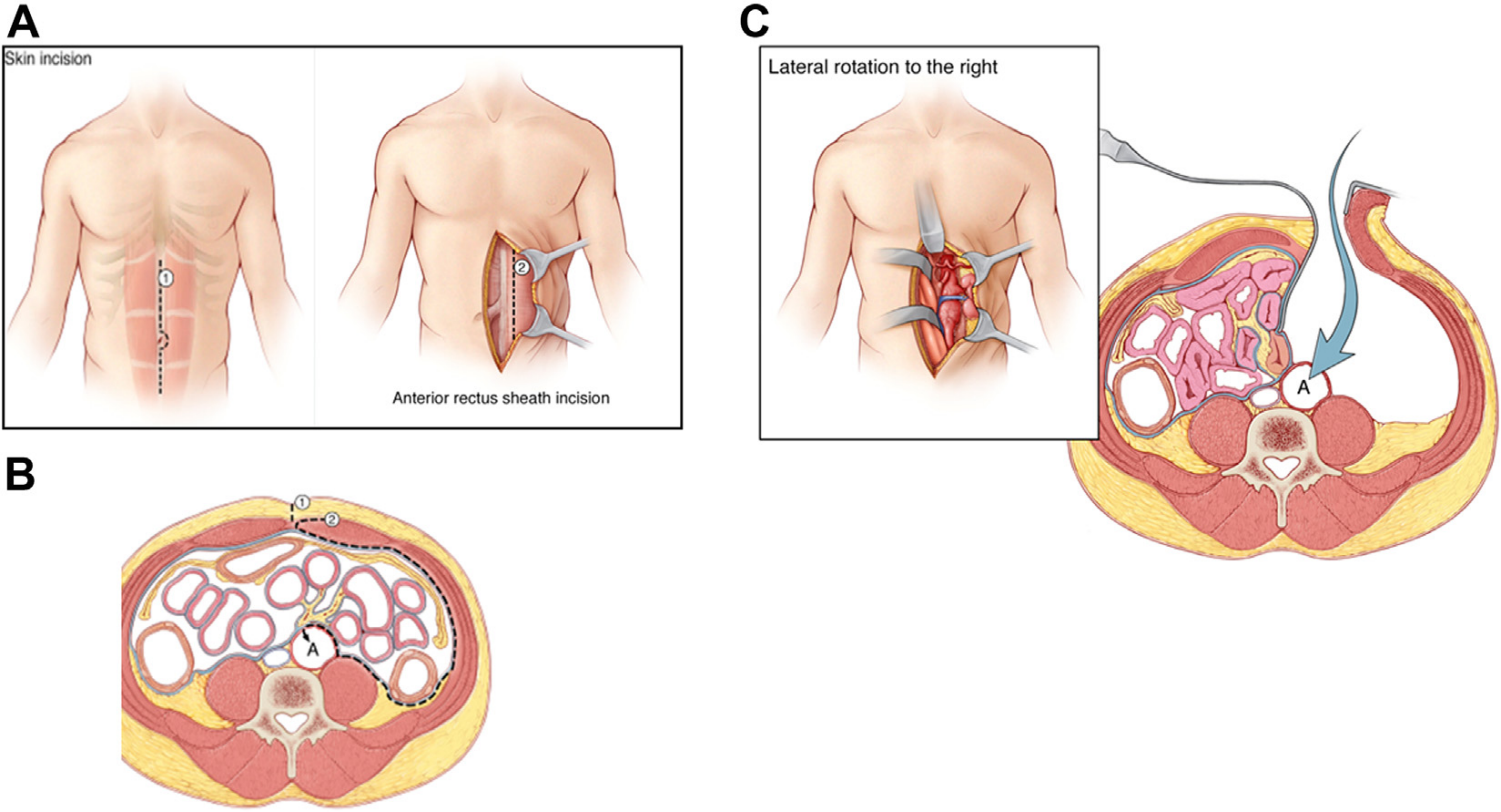
**A,** A xiphoid to pubis midline incision to expose the left anterior rectus sheath 3 cm from the midline. **B,** The left anterior sheath was incised, the rectus muscle was retracted laterally, the pre- and retroperitoneal planes were bluntly developed until the aorta was identified. **C,** The peritoneal sac was mobilized and retracted to the right of the patient to expose the abdominal aorta.

The patient should be positioned in a 30° right lateral position with a roll. During the procedure, the patient will be moved either to a supine position (for the midline incision and initial iliac artery and infrarenal aortic dissection, and proximal and distal anastomoses) or to a 60° right lateral position (during the paravisceral aortic dissection to allow for easier medial mobilization of the peritoneal contents). Using this method, the amount of movement will be only 30° to the left for a supine position or 30° to the right of the patient for a 60° lateral position. With the patient placed in a supine position, a midline, xyphopubic incision is made (Fig 1, *A*, *1*), and the subcutaneous tissue is divided with electrocautery until the linea alba has been identified.

**Fig 2:**
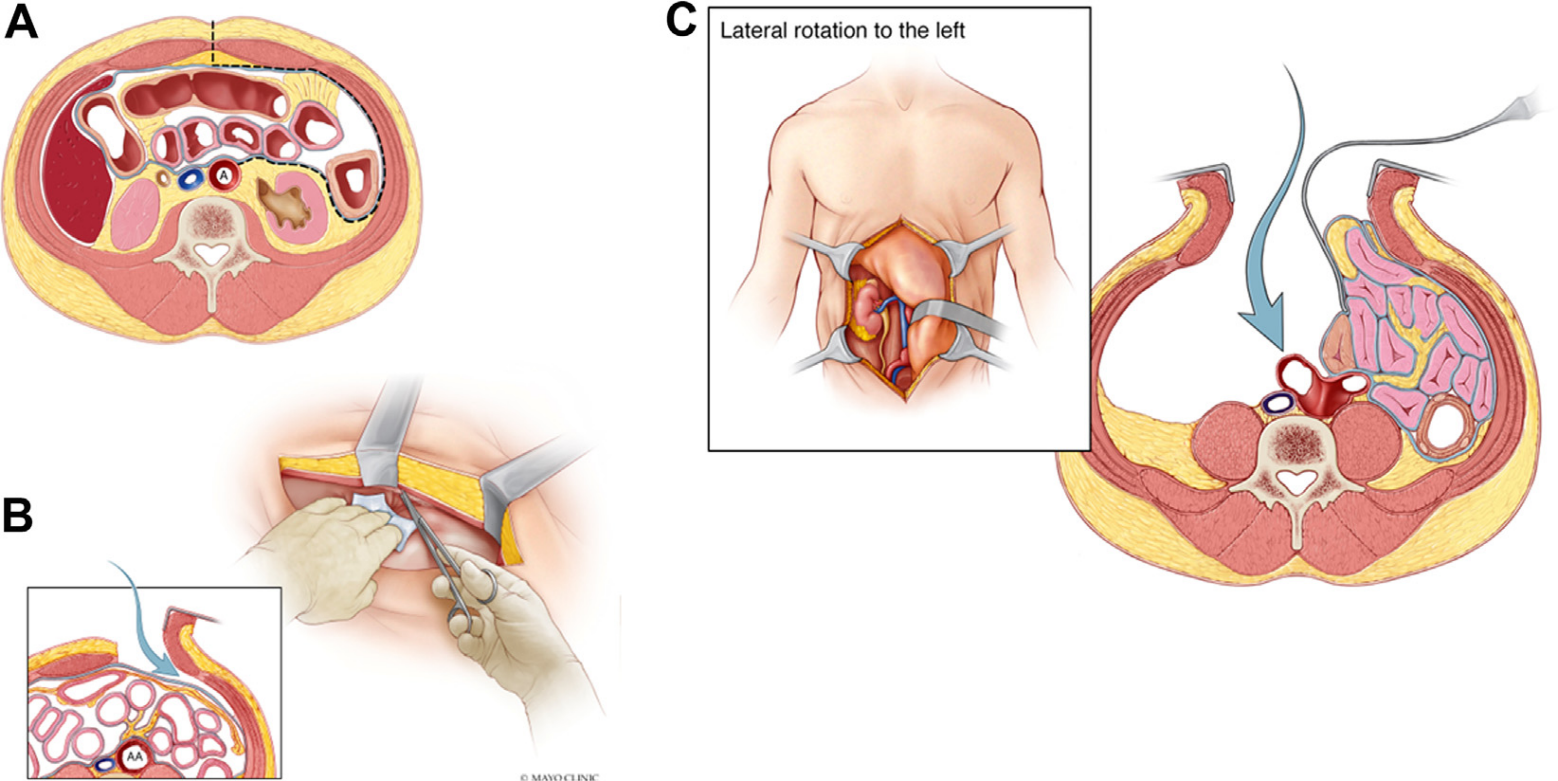
A cross-sectional depiction of the supraumbilical extraperitoneal exposure. **A,** A midline incision was made, the fascia was incised just below the xiphoid process, and the preperitoneal fat was identified. **B,** The peritoneal sac was dissected off the posterior aspect of the left-sided anterior wall with a combination of blunt and sharp dissection. **C,** To access the right common iliac bifurcation, the right anterior rectus sheath was exposed 3 cm from the midline. The right anterior sheath was incised and the rectus muscle retracted laterally. The pre- and retroperitoneal planes were bluntly developed, and the peritoneal sac was retracted toward the left of the patient, exposing the right common iliac bifurcation.

For variant A, the infraumbilical anterior rectal sheath should be freed from the subcutaneous fat for ∼3 cm lateral to the left (Fig 1, *A, 2*), very similar to an anterior spine approach. The fascia should be divided ∼3 cm from the midline and the rectus muscle mobilized laterally. Next, the posterior rectus sheath should be divided ∼2 cm from the midline to provide access to the extraperitoneal space and allow it to be developed (Fig 1, *A* *and* *B*). This process should be continued further to the uppermost extent of the incision. As the sac is mobilized, the gonadal vein will be identified lying adjacent to the peritoneum in a loose fibrous tissue. The peritoneum should be mobilized away from the gonadal vein. This will allow one to develop the correct plane and avoid injuring the left ureter. Next, a table-fixed self-retaining retractor should be used to retract the peritoneal sac (Fig 1, *C*).

For variant B, the supraumbilical linea alba should be incised (Fig 2, *A*). At this level, a fat pad will always be present, separating the abdominal wall from the peritoneum. The fat pad provides an extra layer that will protect the surgeon from accidentally opening the peritoneum. The peritoneum should be dissected off the posterior aspect of the anterior wall using blunt and sharp dissection. Fibrous and fibrovascular strands of the posterior surface of the anterior abdominal wall should be divided sharply with scissors (Fig 2, *B*). It is important to use scissors, because blunt dissection will radially tear the peritoneum. The dissection should continue posteriorly and laterally until the tip of the spleen and Gerota’s fascia can be visualized. Returning to the midline, the linea alba should be gradually divided caudally as the midline peritoneum is freed from it. This process should then be continued to the lowermost extent of the incision and then laterally under the full length of the wound. At this stage, the peritoneum should have been fully mobilized. The lateral conal fascia can be identified by palpation and divided to allow for full mobilization of the peritoneal sac toward the right of the patient. The plane between the peritoneal sac and the kidney, ureter, and gonadal vein should be developed as described.

Variants A and B can be used interchangeably, depending on provider experience. Variant A is similar to anterior spine exposure and could be preferred by those who perform spine exposure in their practice. Variant B resembles a standard transperitoneal approach (TPA), at least for the first part of the exposure, and could be preferred by some surgeons who mostly use a TPA. The only difference is that this approach could be technically difficult, if not impossible, to perform after prior transabdominal surgery, because the fat pad could have been removed and the fascia could have become fused to the peritoneum.

### Aortic exposure

As the left border of the aneurysm is approached, inadvertent opening of the peritoneum can easily occur if the fibrous attachments have not been divided. When the midpoint of the aorta has been reached anteriorly, the inferior mesenteric artery (IMA) pedicle can be identified. If the IMA is chronically occluded or severely diseased, it should be ligated flush to the origin to allow for further mobilization of the peritoneal sac and full exposure of the distal aortic aneurysm. The mobilization should proceed cranially by lifting the third portion of the duodenum, which is covered by the peritoneal sac, up and away from the aorta. To expose the suprarenal aorta, the peritoneum should be dissected from the diaphragm using a combination of sharp and blunt dissection. Again, the peritoneum can be easily opened inadvertently during this part of the dissection, if the fibrous attachments have not been divided sharply. In such cases, the peritoneum should be closed with 3-0 Vicryl suture (Ethicon Inc, Johnson & Johnson, New Brunswick, NJ). By freeing the peritoneum from the entire left side of the diaphragm and retracting the peritoneum sac and its contents to the right of the patient, the surgeon will have full exposure of the entire aorta, including several centimeters of the supraceliac aorta. However, if supraceliac aortic exposure is not needed, the peritoneum will not need to be freed from the diaphragm.

### Exposure of right iliac artery bifurcation

If exposure of the right common iliac bifurcation is needed, it should be obtained at the beginning of the procedure, after the midline incision is deepened down to the linea alba and while the patient is level. Control of the right iliac artery bifurcation can be obtained by dividing the anterior rectus sheath, 3 cm to the right of the midline, similar to the method used for left exposure. The rectus muscle should be mobilized laterally and the retrorectus, extraperitoneal space bluntly developed (Fig 2, *C*). Next, the right ureter should be identified and protected. The common, external, and internal iliac arteries should be dissected free and controlled with vessel loops. This approach adds a second incision to the anterior sheath of the rectus muscle, to the right of the linea alba.

### Pain management

We developed a pain control protocol for abdominal vascular surgery in conjunction with our anesthesia colleagues:

• Preoperatively: oral acetaminophen (Tylenol; McNeil Consumer Healthcare, Johnson & Johnson), 1000 mg, and celecoxib, 400 mg (200 mg for patients aged ≥65 years; withheld for patients with significant renal or hepatic dysfunction)
• Intraoperatively: intravenous ketamine, 10 mg each hour, a transverse abdominis plane block via an injection of a bupivacaine liposome injectable suspension (Exparel; Pacira Pharmaceuticals, Parsippany-Troy Hills, NJ), and intravenous acetaminophen 1000 mg (if >6 hours since previous dose)
• Postoperatively: scheduled oral acetaminophen 1000 mg, intravenous ketorolac (Ketorolac) 15 mg (if no concerns exist for bleeding and estimated glomerular filtration rate >60 mL/min/1.73 m^2^), intravenous methocarbamol (Methocarbamol) 1000 mg three times daily, ice to the surgical incision, oral oxycodone as needed, and intravenous hydromorphone (Dilaudid; Purdue Pharma, LP, Stamford, CT) as needed

## Case report

The institutional review board determined that the present study was exempt from the requirement for approval. The four patients provided written informed consent for the report of their case details and imaging studies. None of the four patients had been candidates for endovascular repair for several reasons, including the extent of the atherosclerotic occlusive disease, presence of aortic thrombus in the paravisceral aorta, need to preserve flow to the IMA and/or hypogastric artery, and/or an inadequate infrarenal neck (in the case of aneurysm). The preoperative imaging studies included computed tomography angiography of the abdomen and pelvis, bilateral carotid artery ultrasound, a cardiac stress test, and pulmonary function tests to determine the patients’ clearance for surgery. Active smokers were required to have abstained for ≥4 weeks preoperatively. A bifurcated, Dacron graft was sutured to the aorta with a 3-0 Prolene running suture (Ethicon Inc) in an end-to-end fashion in all four cases.

### Patient 1

**Fig 3:**
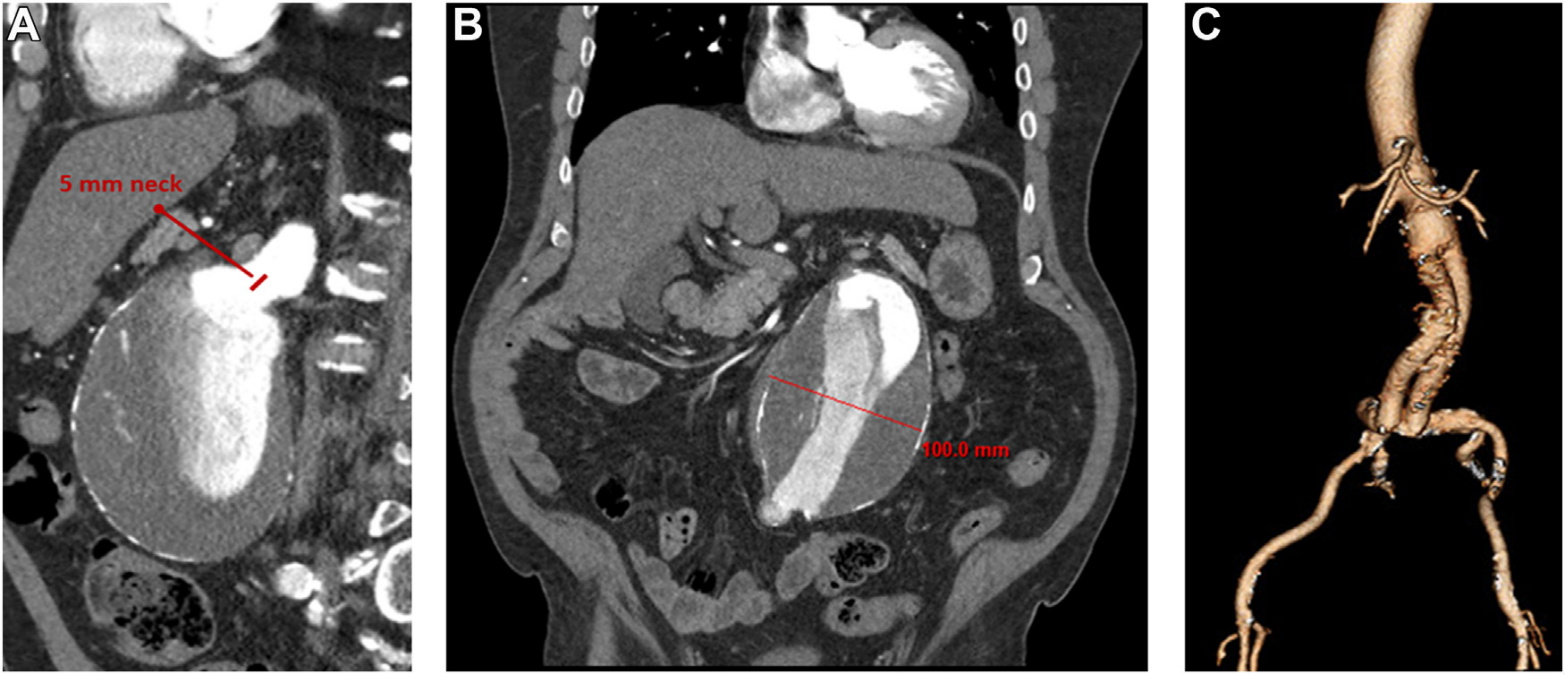
**A,** Sagittal computed tomography view demonstrating a short, 5-mm-long, angulated abdominal aortic aneurysm neck. **B,** Coronal view showing the maximal diameter of the aneurysm was 100 mm. **C,** Three-dimensional computed tomography scan reconstruction at 1 year postoperatively demonstrating widely patent anastomoses.

A 68-year-old man had been incidentally found to have an asymptomatic, 10-cm infrarenal aortic aneurysm with a short and angulated neck (5 mm; Fig 3, *A* *and* *B*). He was a current smoker with the following comorbidities: hypertension, hyperlipidemia, diabetes mellitus type 2, chronic obstructive pulmonary disease requiring continuous positive airway pressure, and obesity. He had no family history of aneurysmal disease.

We decided to perform open repair with an aortic bi-iliac bypass. The MRA approach was chosen to allow access to the supramesenteric aorta. We believe that the RPA (regardless of whether through a lateral flank or midline incision) will provide better ease at exposing the paravisceral aorta compared with the TPA. The patient underwent repair with suprarenal clamping and juxtarenal proximal anastomosis with a Dacron graft. The operative time was 10 hours, 6 minutes, and the blood loss was 2500 mL.

Postoperatively, the patient’s pain was well controlled. He had a return of bowel function and started a clear liquid diet on postoperative day (POD) 2. The patient had appropriate urine output, with a creatinine peak of 1.3 mg/dL, which had returned to baseline (0.9 mg/dL) at discharge on POD 5. His postoperative analgesic medications included scheduled acetaminophen and oxycodone and hydromorphone as needed, for a total of 300 morphine equivalents, during his hospitalization. The patient had returned to his baseline activities at 3 months postoperatively. At the 1-year follow-up, the graft was widely patent (Fig 3, *C*).

### Patient 2

**Fig 4:**
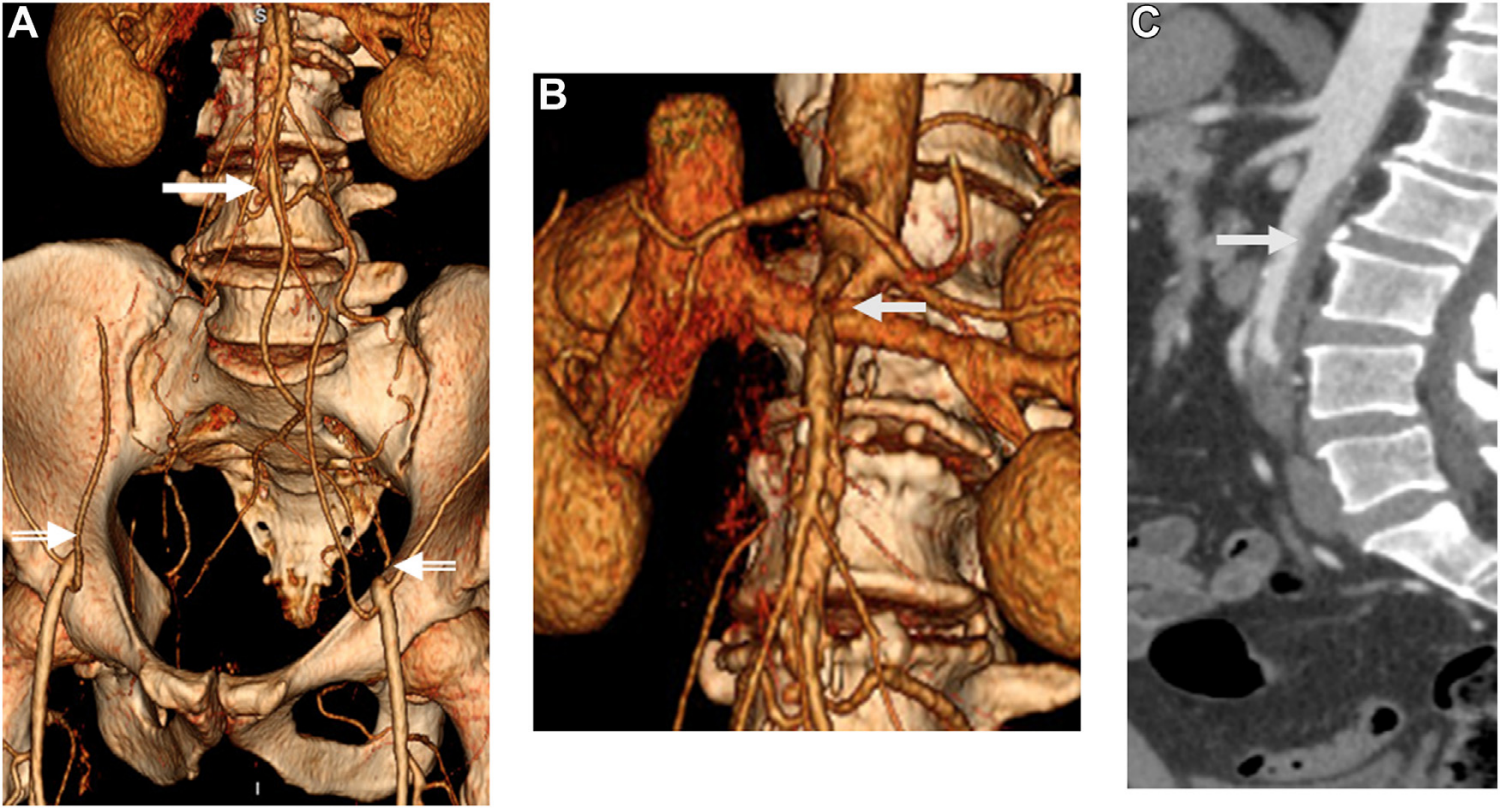
**A,** Three-dimensional computed tomography reconstruction demonstrating aortic occlusion starting just distal to the takeoff of a large inferior mesenteric artery (IMA; *solid white arrow*). The common, internal, and external iliac arteries were also chronically occluded. The distal external iliac arteries were reconstituted via the inferior epigastric artery, bilaterally (*interrupted white arrows*). **B,** The superior mesenteric artery (SMA) had severe stenosis at 3 cm from its origin. **C,** A sagittal computed tomography scan showing extensive, aortic soft plaque starting at the level of the SMA and causing significant stenosis.

A 50-year-old man had presented with bilateral, short-distance, gluteal and thigh claudication secondary to occlusion of the aorta distal to the takeoff of a large IMA and occlusion of the bilateral common, external, and internal iliac arteries (Fig 4, *A*). An aortic posterior laminar thrombus had extended up to the level of the superior mesenteric artery (SMA) with a high degree of stenosis of the SMA (Fig 4, *B* *and* *C*). The patient denied postprandial symptoms. His comorbidities included hypertension, hyperlipidemia, and current smoking.

**Supplementary Fig 1:**
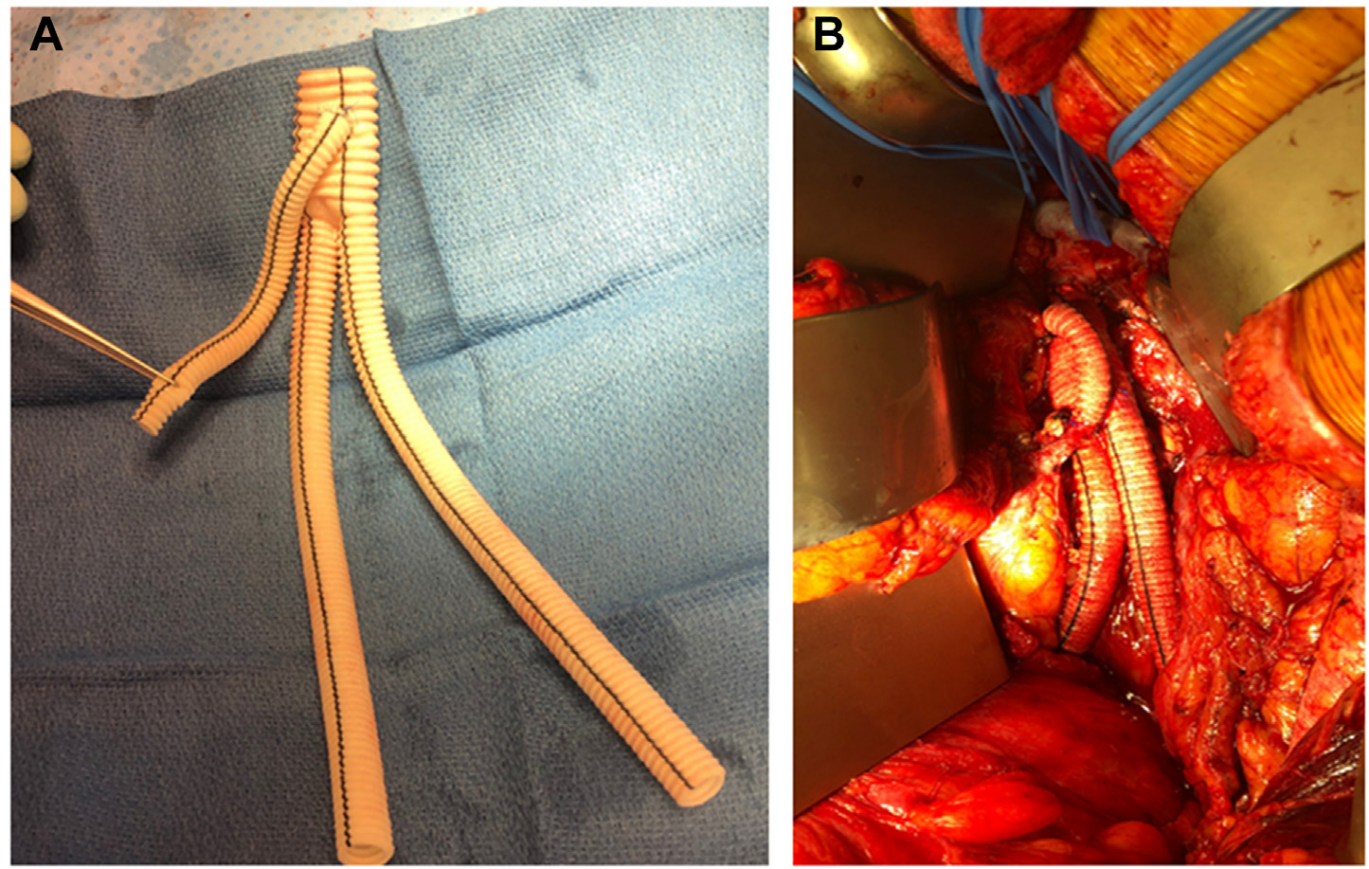
**A,** The Dacron graft was configured as an aortobifemoral graft with a jump graft to the inferior mesenteric artery (IMA). **B,** Completion intraoperative imaging study demonstrating end-to-end anastomosis of the jump graft to the IMA.

**Supplementary Fig 2:**
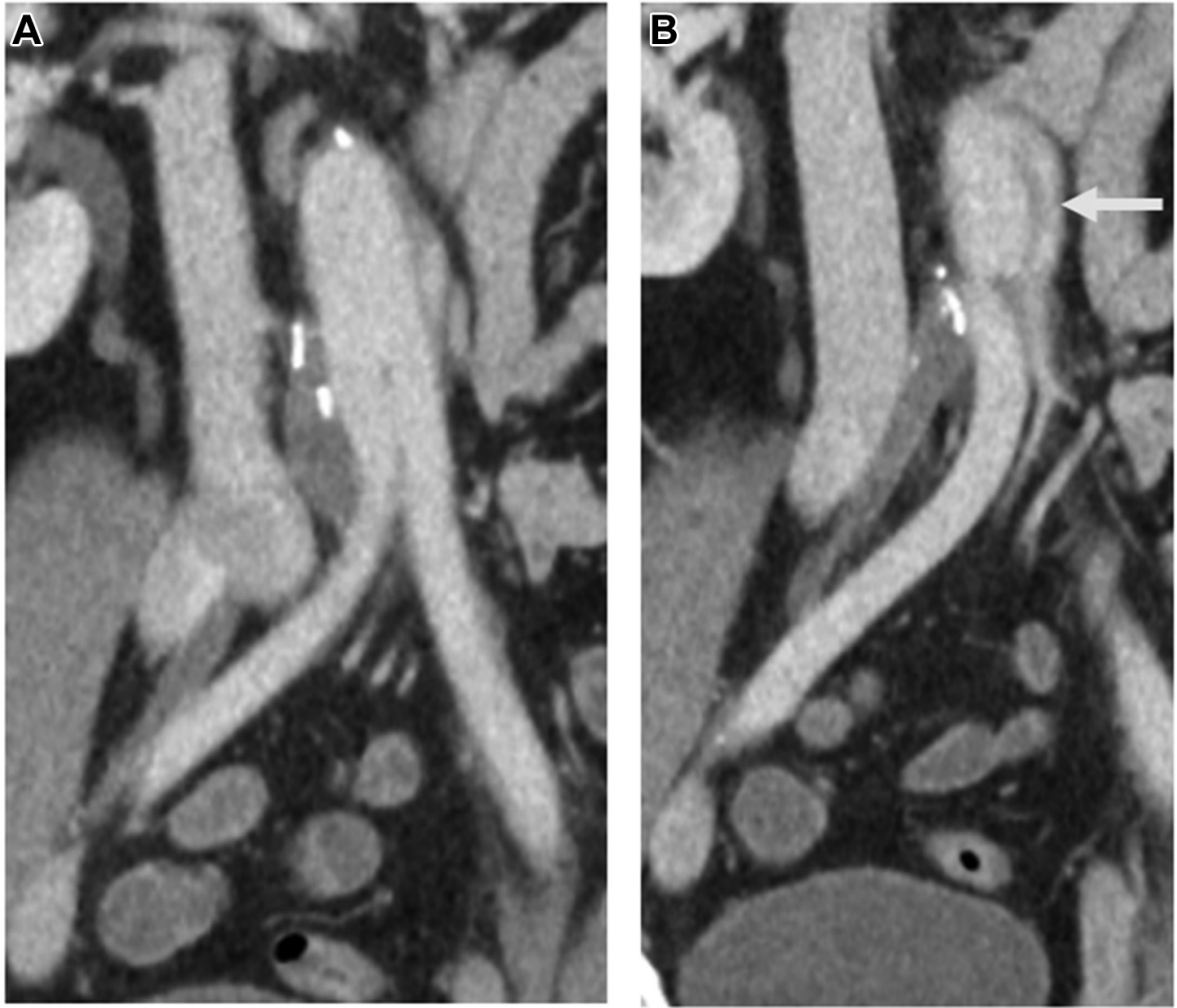
Postoperative coronal computed tomography scan at 3 months demonstrating patency of the aortobifemoral graft (**A**) and inframesenteric artery jump graft (**B**; *solid white arrow*).

He underwent aortobifemoral bypass with a jump graft to the IMA to minimize the risk of mesenteric ischemia (Supplementary Fig 1). The patient had required a supraceliac clamp where the aorta was free of laminar thrombus. The operative time was 12 hours, 3 minutes, and the blood loss was 1250 mL. Intraoperatively, the patient had developed a fever and an increasing oxygen requirement. The patient was kept intubated postoperatively. Computed tomography of chest obtained on POD 1 demonstrated right-sided pneumonia. Subsequent investigation revealed that the patient, unknown to the healthcare team, had had complaints of a productive cough for 1 week before surgery and had not been compliant with smoking cessation. The patient had required prolonged intubation but was ultimately discharged to a rehabilitation facility at 3 weeks after surgery. At the 3-month follow-up, the patient was progressing well. At 1 year, the grafts were widely patent (Supplementary Fig 2).

### Patient 3

**Supplementary Fig 3:**
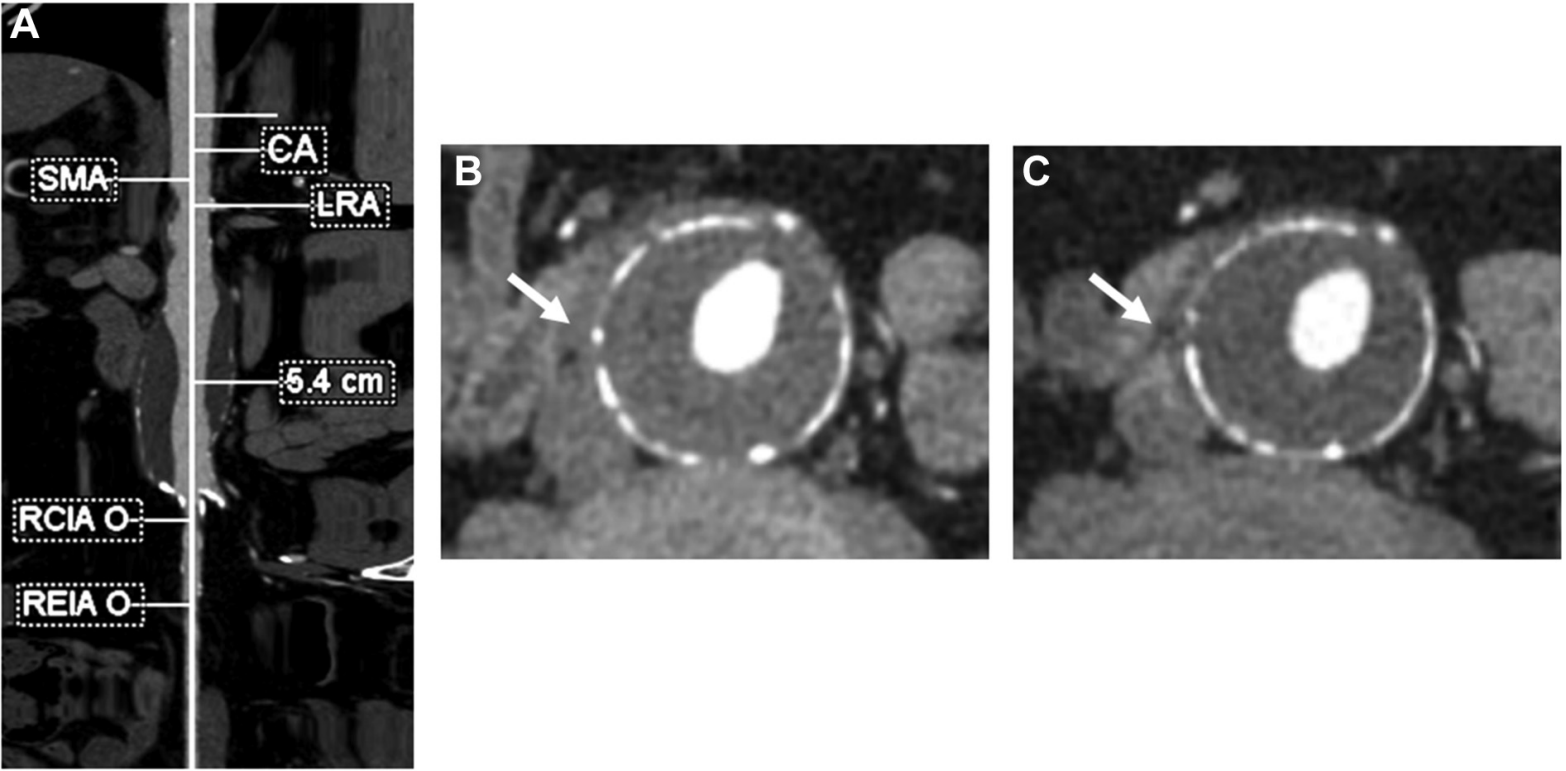
**A,** Central line measurement showing the origin of the visceral aortic branches. The aneurysm started at the level of the left renal artery (juxtarenal) and measured 5.4 cm in the largest diameter. **B,** Coronal computed tomography scan demonstrating aortic wall thickness that was concerning for an inflammatory aneurysm. **C,** After 3 months of steroid administration, partial resolution of the inflammation was seen. *CA,* Carotid artery; *LRA,* left renal artery; *RCIA,* right common iliac artery; *REIA,* right external iliac artery; *SMA,* superior mesenteric artery.

A 63-year-old man had presented with a symptomatic (tender to palpation), 5.4-cm inflammatory juxtarenal abdominal aortic aneurysm. He also complained of symptoms of claudication secondary to right common and external iliac stenosis. No ureteral obstruction was found. After the diagnosis, he underwent a 3-month course of steroid therapy without significant improvement in the overall size of the aneurysm but slight decrease in the aortic wall thickening (Supplementary Fig 3, *A* *and* *B*). Patient had continued to have abdominal tenderness to palpation over the aneurysm. His comorbidities included hypertension, hyperlipidemia, and a history of smoking.

**Fig 5:**
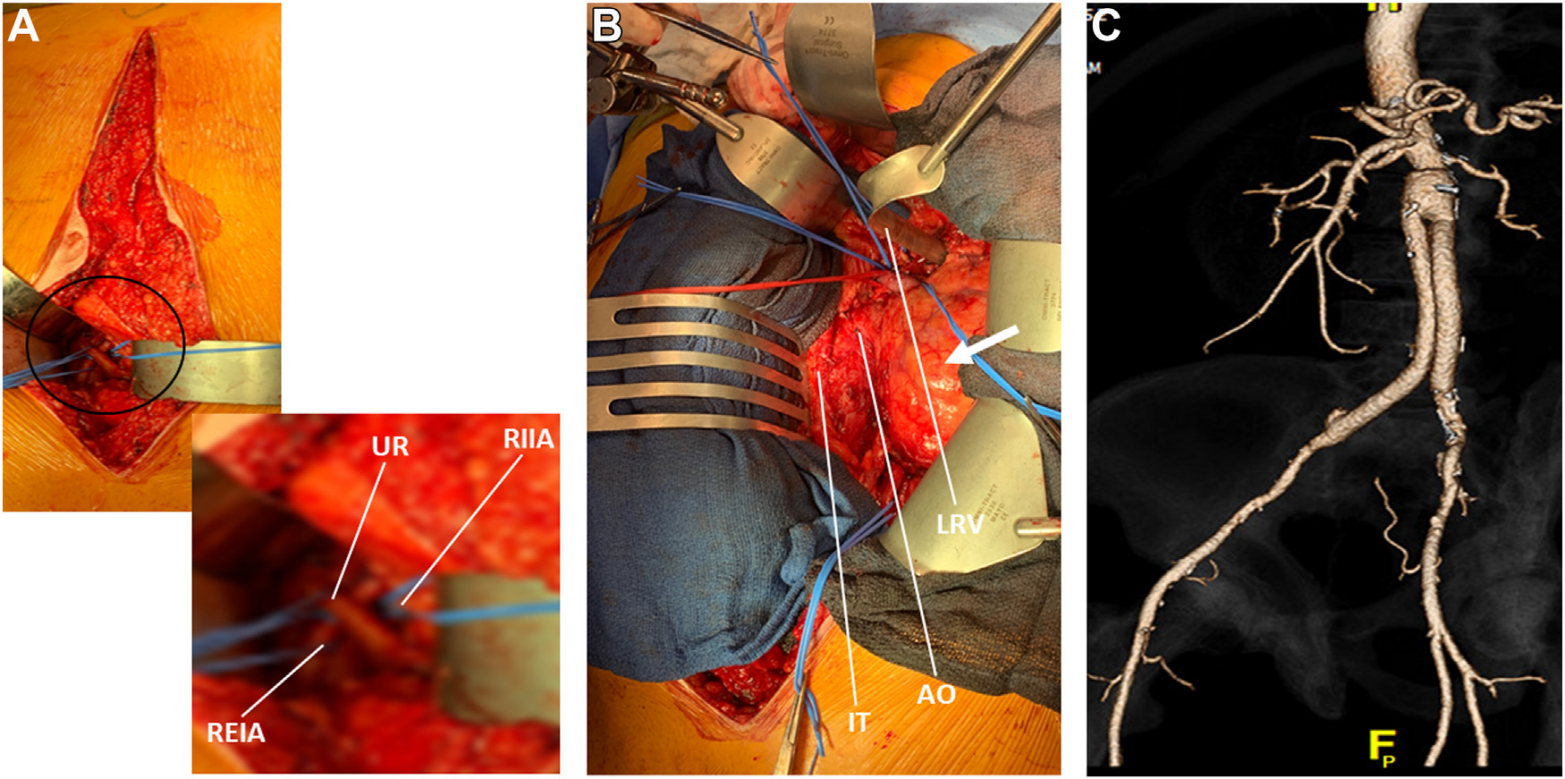
**A,** Surgical control of the right common iliac artery bifurcation. The right anterior rectus sheath was divided 3 cm from the midline, the rectus muscle was retracted laterally, and the pre- and retroperitoneal planes were developed bluntly until the iliac bifurcation had been identified medial to the psoas muscle. **B,** The paravisceral aorta was exposed and all mesorenal branches were controlled with vessel loops. **C,** Three-dimensional computed tomography scan at 1 year postoperatively showing a widely patent graft, visceral branches, and anastomoses. *REIA,* right external iliac artery; *RIIA,* right internal iliac artery; *UR,* ureter.

With the challenges due to the inflammatory nature of the aneurysm, preoperative ureteral stents were placed, and a midline RPA was chosen to achieve control of the right common iliac bifurcation. The right retroperitoneal space was developed first through a midline incision by incising the right anterior rectus sheath, 3 cm lateral to the midline. Once the right iliac bifurcation was controlled (Fig 5, *A*), the left retroperitoneal plane was developed by incising the left anterior rectus sheath, 3 cm from the midline (Fig 5, *B*). The patient required a supraceliac clamp, where the aorta was free from dense inflammation, and an aortic-to-right external and left common iliac artery bypass with a Dacron graft was performed. The proximal anastomosis was sutured in a juxtarenal position after the paravisceral aorta had been cleared of thrombus. The operative time was 7 hours, 44 minutes, and the blood loss was 2205 mL.

Postoperatively, the patient's pain was well controlled, with an early return of bowel function. The patient started a clear liquid diet on POD 2 with subsequent advancement to a normal diet and was discharged home on POD 5. His postoperative analgesic medications included scheduled acetaminophen and oxycodone and hydromorphone as needed, for a total of 312 morphine equivalents during his hospitalization. At 1 year of follow-up, the patient had returned to his normal baseline activities, his symptoms of claudication had resolved, and computed tomography angiography showed the Dacron graft was widely patent (Fig 5, *C*).

### Patient 4

**Supplementary Fig 4:**
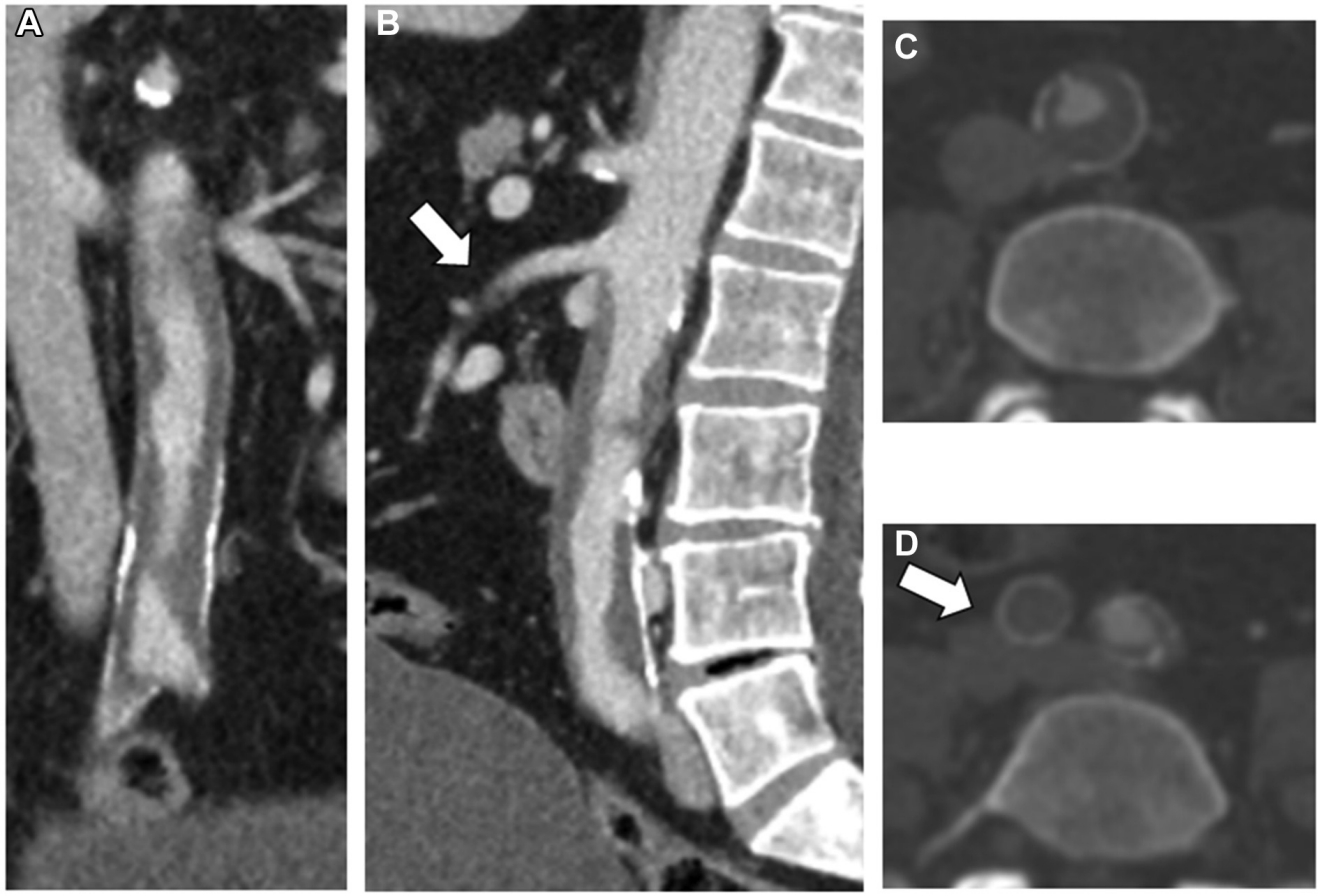
Coronal **(A)** and sagittal **(B)** computed tomography scans demonstrating a large amount of aortic soft plaque extending from the level of the superior mesenteric artery (SMA) to the iliac arteries, bilaterally. **C,** The soft plaque had caused a significant reduction in the aortic lumen. **D,** The right common iliac artery was chronically occluded.

**Supplementary Fig 5:**
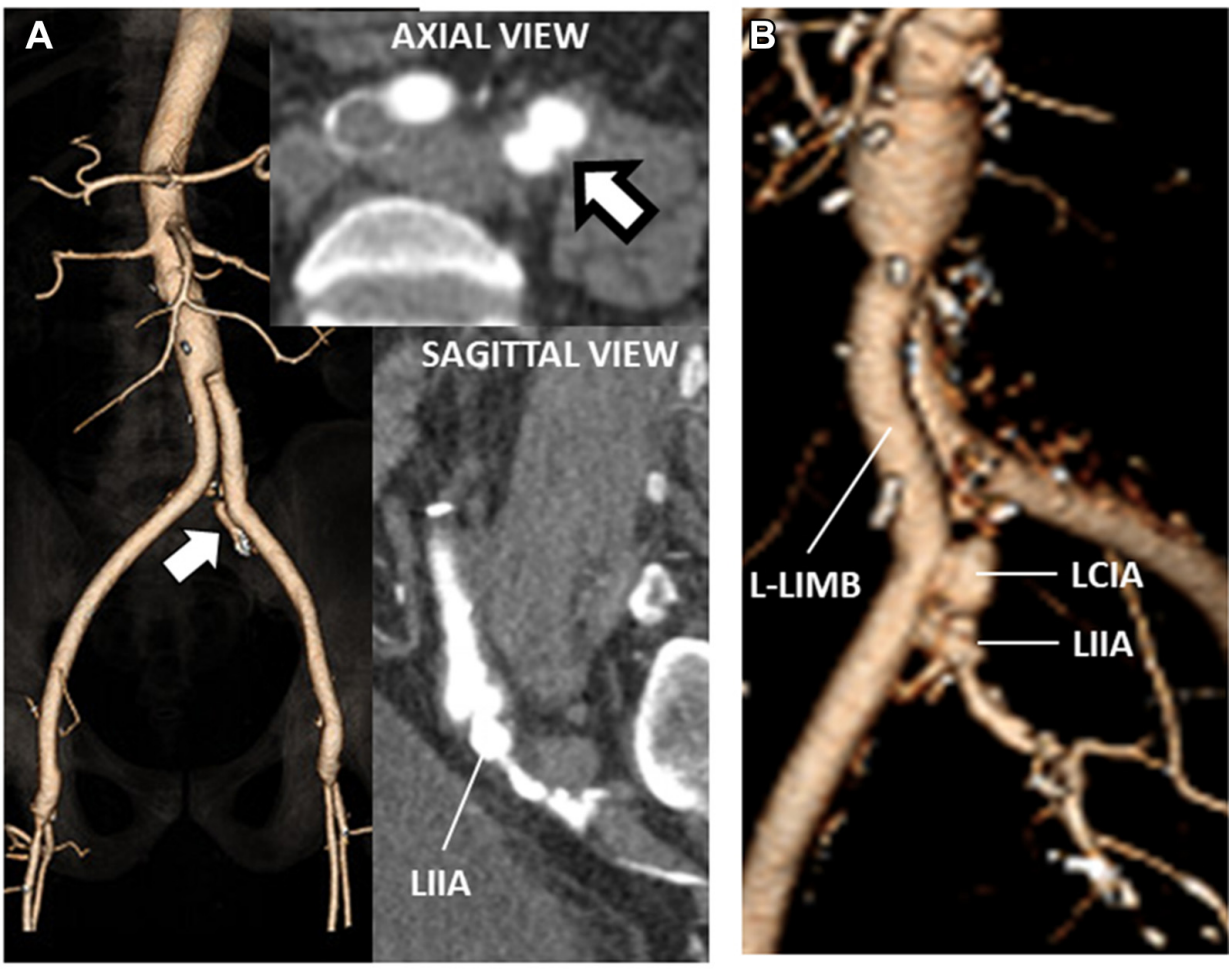
Coronal **(A)** and sagittal **(B)** view computed tomography scans for the 3 months of follow-up showing a widely patent graft and patent side-to-side left limb-to-left common iliac artery anastomosis to provide flow to the left hypogastric artery. *L-LIMB,* Left limb; *LCIA,* left common iliac artery; *LIIA,* left internal iliac artery.

A 69-year-old man had presented with bilateral, short-distance, gluteal and thigh claudication secondary to occlusion of the right common iliac, bilateral external iliac, and right internal iliac arteries, with a large amount of thrombus present in the pararenal aorta (Supplementary Fig 4). The IMA was chronically occluded. The patient had undergone percutaneous, left renal mass ablation 3 months before the present surgery. His comorbidities included hypertension, hyperlipidemia, and recent smoking. Because of the extent of the thrombus, a supra-SMA clamp was required, making a RPA preferable. Intraoperatively, an extensive fibrous–inflammatory reaction was found at the level of the pararenal aorta, limiting exposure of the supra-SMA aorta. We decided to position the clamp between the renal arteries. After the interrenal clamp had been placed, the aorta was opened longitudinally up to the left renal artery, and the thrombus was cleared. The aortotomy was closed with 3-0 running Prolene suture (Ethicon Inc). The clamp was then moved to the infrarenal aorta (renal ischemia time, 13 minutes), the aorta was transected 3 cm below the left renal artery, and a bifurcated Dacron graft was anastomosed to the infrarenal aorta. In addition, a side-to-side anastomosis between the distal left common iliac artery and the left limb of the graft was performed to provide flow to the left iliac artery (Supplementary Fig 5). The operative time was 10 hours, 43 minutes, and the blood loss was 4000 mL. The patient started a clear liquid diet on POD 2 with subsequent advancement to a normal diet and was discharged home on POD 5. His postoperative analgesic medications included scheduled acetaminophen and methocarbamol and oxycodone and hydromorphone as needed, for a total of 110 morphine equivalents during his hospitalization. At the 1-month and 3-month follow-up, the patient was progressing well (Supplementary Fig 5).

## Discussion

In the present report, we have summarized our early experience using a midline RPA, which we have further developed to treat complex aortoiliac disease. We found that this approach provides excellent exposure to the paravisceral aorta, right renal artery, and right iliac bifurcation, with appropriate patient outcomes. In the present report, we have highlighted the main procedural steps of the MRA with the intent of aiding surgeons interested in adopting this approach for abdominal aorta access.

The two most common approaches to the abdominal aorta have been the TPA and the RPA through a left flank incision. At our institution, the TPA has remained the most common choice for open repair of abdominal aortic aneurysms.^3^ Whether that preference was truly data driven or because most surgeons have been trained in this approach is difficult to discern. The TPA has the advantages of being well-known to all vascular and general surgeons, allows for evaluation of bowel viability, gives access to the both the iliac bifurcation and the suprarenal aorta, and can be converted to retroperitoneal access by performing a left medial visceral rotation. The disadvantages include the occurrence of postoperative ileus and limited access to the supramesenteric aorta. In addition, the TPA might not be suitable for patients with a hostile abdomen, a horseshoe kidney, or an inflammatory aneurysm. At our institution, the RPA through a flank incision has been preferred for a hostile abdomen, a horseshoe kidney, and an inflammatory aneurysm and seems to have been associated with a shorter duration or absence of ileus postoperatively.^4^, ^5^, ^6^ However, the TPA requires division of the lateral abdominal wall muscles with frequent muscular and/or flank bulging from muscle denervation and significant postoperative pain.^7^^,^^8^ Furthermore, the TPA does not allow for exposure of the right iliac bifurcation and provides limited visualization of the right renal artery.

Limited data of the MRA are available, and it has only been described to treat infrarenal aortic disease.^7^, ^8^, ^9^, ^10^ The MRA has been shown to provide many advantages; however, it has not been included in many vascular surgery training programs. A few groups have compared the MRA with the TPA in terms of operative details, gastrointestinal complications, and wound complications.^7^^,^^9^^,^^11^ The two groups showed no statistically significant differences in the aortic clamping or total operation times. However, the MRA was associated with a significantly shorter return to bowel function and shorter hospital stay statistically.^7^^,^^9^ Furthermore, the incidence of wound complications was decreased with the MRA.^7^^,^^11^

We found that use of the MRA overcame some of the disadvantages of the RPA but required a longer exposure and longer total operative time (mean, 9 hours and 40 minutes). The longer procedural time had resulted, in part, from the learning curve in our early experience. However, our patients demonstrated appropriate postoperative progress with graft patency at 1 year. In addition to our operative approach, we believe the use of appropriate perioperative pain control complimented the overall patient hospital course and convalescence. It is well-known that adequate pain management is crucial for postoperative recovery because poorly controlled pain can increase patients’ morbidity and mortality.^12^ Traditionally, opioids have been the main drugs for postoperative pain control after open vascular surgery.^13^ However, consistent with the efforts to develop opioid-sparing protocols for postoperative pain control, we developed a multimodal pain control protocol in conjunction with the anesthesiology department for open vascular abdominal surgery. The use of this protocol has significantly decreased the use of opioids compared with the historical data within our institution.

## Conclusions

In our initial experience, the MRA provided excellent exposure to the paravisceral aorta for complex aortic reconstruction, allowed for exposure of the right renal artery and right iliac artery bifurcation, and avoided division of the lateral abdominal wall muscles and the associated flank bulge and pain. In addition, the use of the MRA seemed to be associated with an early return of bowel function. We believe that the MRA should be considered for elective cases for which the RPA would otherwise be indicated.

## Author Contributions

Conception and design: EB, JB, TT

Analysis and interpretation: EB, BF, NS, TC, TT

Data collection: EB, CG

Writing the article: EB, JB, TT

Critical revision of the article: EB, JB, CG, BF, NS, TC, TT

Final approval of the article: EB, JB, CG, BF, NS, TC, TT

Statistical analysis: Not applicable

Obtained funding: Not applicable

Overall responsibility: TT
